# Prognostic model based on six PD-1 expression and immune infiltration-associated genes predicts survival in breast cancer

**DOI:** 10.1007/s12282-022-01344-2

**Published:** 2022-03-01

**Authors:** Shen Junjun, Zhuang Jing, Pu Jie, Chu Jian, Pan Yuefen, Han Shuwen

**Affiliations:** 1 Department of Medical Oncology, Huzhou Central Hospital, Affiliated Central Hospital Huzhou University, No. 1558, Sanhuan North Road, Wuxing District, Huzhou, 313000 Zhejiang China; 2 Graduate School of Medical College of Zhejiang University, No. 268 Kaixuan Road, Jianggan District, Hangzhou, 310029 Zhejiang China; 3 Department of Oncology, Huzhou Central Hospital, Affiliated Central Hospital Huzhou University, No. 1558, Sanhuan North Road, Wuxing District, Huzhou, 313000 Zhejiang China; 4 Graduate School of Nursing, Huzhou University, No. 1 Bachelor Road, Huzhou, 313000 Zhejiang China; 5 Graduate School of Second Clinical Medicine Faculty, Zhejiang Chinese Medical University, No. 548 Binwen Road, Binjiang District, Hangzhou, 310053 Zhejiang China; 6 Department of Oncology, Huzhou Central Hospital, Affiliated Central Hospital Huzhou University, No. 1558, Sanhuan North Road, Wuxing District, Huzhou, Zhejiang China

**Keywords:** Breast cancer, Programmed cell death-1, Immune infiltration, Prognosis, Immunotherapy

## Abstract

**Background:**

The prognosis of breast cancer (BC) was associated with the expression of programmed cell death-1 (PD-1).

**Methods:**

BC-related expression and clinical data were downloaded from TCGA database. PD-1 expression with overall survival and clinical factors were investigated. Gene set variation analysis (GSVA) and weighted gene correlation network analysis were performed to investigate the PD-1 expression-associated KEGG pathways and genes, respectively. Immune infiltration was analyzed using the ssGSEA algorithm and DAVID, respectively. Univariate and multivariable Cox and LASSO regression analyses were performed to select prognostic genes for modeling.

**Results:**

High PD-1 expression was related to prolonged survival time (*P* = 0.014). PD-1 expression status showed correlations with age, race, and pathological subtype. ER- and PR-negative patients exhibited high PD-1 expression. The GSVA revealed that high PD-1 expression was associated with various immune-associated pathways, such as T cell/B cell receptor signaling pathway or natural killer cell-mediated cytotoxicity. The patients in the high-immune infiltration group exhibited significantly higher PD-1 expression levels. In summary, 397 genes associated with both immune infiltration and PD-1 expression were screened. Univariate analysis and LASSO regression model identified the six most valuable prognostic genes, namely IRC3, GBP2, IGJ, KLHDC7B, KLRB1, and RAC2. The prognostic model could predict survival for BC patients.

**Conclusion:**

High PD-1 expression was associated with high-immune infiltration in BC patients. Genes closely associated with PD-1, immune infiltration and survival prognosis were screened to predict prognosis.

**Supplementary Information:**

The online version contains supplementary material available at https://doi.org/10.1007/s12282-022-01344-2.

## Introduction

Breast cancer (BC) is the most common malignancy in women that ranks as the main cause of cancer-related deaths in women [1]. According to the 2020 Global Cancer Statistics, BC accounted for 11.7% of the 19.3 million new cancer cases and 6.9% of the 10 million cancer-related deaths in women [2]. With the progress in early diagnosis and synthetic therapeutic strategies, BC prognosis improved, with a 5-year survival rate of over 80% for non-metastatic BC [3]. However, 20–30% of the BC cases demonstrate a metastatic disease following diagnosis and primary tumor treatment, with a 25% 5-year survival rate and approximately 90% of cancer-related deaths attributed to metastasis [4]. In metastatic BC, the curative goals are prolonging the survival and maintaining the quality of life [5, 6]. Immunotherapy is one of the current treatment methods for malignant tumors, which is expected to bring survival benefits to BC patients [7].

Tumor cell immune escape is the major reason responsible for malignant tumor treatment difficulties. Immunity in the human body is mainly mediated by T cell-related cellular immunity, and T cell activation to develop the immune response depends on the second signal delivered from co-stimulatory molecules [8, 9]. Programmed cell death-1 (PD-1, also named PDCD1) and its ligand PD-L1 (also named CD274) belong to the CD28/B7 family, functioning as co-stimulatory molecules involved in the regulation of immune responses [10]. PD-1 is an inhibitory receptor induced by the activation of the immunoglobulin superfamily and is mainly expressed in T cells, whereas its ligand PD-L1 is mainly expressed in antigen-presenting cells [11]. When PD-L1 and PD-1 interact, the tumor T lymphocyte immune response is suppressed, so that tumor cells can escape the immune response [10]. Specific antibodies blocking the PD-1/PD-L1 axis could enhance the tumor T lymphocyte immune function and promote immune activity [12].

An increasing number of studies have shown the effect of the PD-1/PD-L1 blockade in the improvement of the adverse outcome in advanced malignant melanoma [13] and other solid tumors, such as non-small cell lung cancer [14], bladder cancer [15], or head and neck cancer [16]. BC is known for being weakly immunogenic with a lower mutational load than other tumor types, and therefore BC, has not experienced the advances in immunotherapy yet [17]. Noske et al. demonstrated that the tumor-infiltrating lymphocyte density in BC showed significant correlations with PD-1 expression in the tumor cells and PD-1/PD-L1 expression in the immune cells, and PD-1-positive immune cells in triple-negative BC (TNBC) were related to a significantly favorable disease-free survival [18]. Recently, the PD-1/PD-L1 blockade has also been evaluated in BC, especially in TNBC, and promising results could be observed [17]. For example, Schmid et al. suggested that in early TNBC, a higher proportion of patients showed pathological complete response among those receiving pembrolizumab (humanized monoclonal anti-PD1 antibody) combined with neoadjuvant chemotherapy (64.8%) than those receiving placebo combined with neoadjuvant chemotherapy (51.2%) [19]. In the KEYNOTE-355 phase III clinical trial, pembrolizumab plus chemotherapy presented a significant and clinically meaningful improvement in the progression-free survival compared to placebo plus chemotherapy among patients with metastatic TNBC with a combined positive score of ≥ 10 [20]. These results suggest that the PD-1/PD-L1 blockade is an emerging novel therapeutic strategy for BC. However, its underlying regulatory mechanisms are still unclear.

Therefore, based on the expression and clinical data in TCGA database, we investigated PD-1 expression, prognostic value, and clinical correlations. Moreover, using weighted gene correlation network analysis (WGCNA) and the LASSO regression algorithm, PD-1 expression and tumor-infiltrating immune cell-associated biomarkers were identified to establish a prognostic risk model. Effective biomarkers and accurate prognosis are critical for healthcare decision evaluation and anti-PD-1 therapeutic response optimization. Prognostic models are reportedly available to stratify patients with different prognoses and predict overall survival. Our study illustrates novel aspects of PD-1 with potential relevance of biological regulation and immunotherapy response stratification.

## Methods

### PD-1 expression with survival analysis and clinical factors

The log2(fpkm+1) RNA-Seq data, as well as the clinical and survival data of BC, in TCGA database were acquired from the UCSC Genome Browser. The genes were annotated according to the human gene annotation file (version 22) provided by GENCODE. We extracted the data on PD-1 and PD-L1 expression. PD-1 and PD-L1 expression with overall survival and PD-1 expression with clinical factors, including age, race, tumor stage, pathologic T/N/M stage, and estrogen receptor (ER), her2 receptor, and progesterone receptor (PR) status were investigated using the survival package (version 2.42-6) and ggstatsplot (version 0.6.5) in R. K–M survival analysis among BC subtypes, such as ER+/HER2−, HER2+ , TNBC was performed using the survival package in R.

**Table:** 

	HER-2	ER	PR
HER-2+	+	−/ +	−/ +
TNBC	−	−	−
ER+ /HER2−	−	+	−/ +

### Identification of PD-1-associated genes

GSVA analysis was conducted to calculate the enrichment score of each KEGG pathway using the GSVA software (version 1.36.2) [21]. High- and low-expression PD-1 groups were divided using the limma package (version 3.10.3) [22] in R according to median value. The classical Bayesian method provided by the limma package (version 3.10.3) in R was used to screen DEGs associated with PD-1. Weighted gene correlation network analysis (WGCNA) using the WGCNA package (version 1.61) [23] was performed to select key correlating modules with PD-1 expression and PD-1-associated genes.

### Identification of immune infiltration-associated genes

Based on the single sample GSEA (ssGSEA) algorithm, the enrichment score of each immune cell in the sample was calculated using the GSVA package (version 1.36.2) in R. Unsupervised clustering was then performed using the heatmap package (version 1.0.12), to assign samples into high- and low-infiltration groups. Immune infiltration-associated genes were screened using the limma package in R.

### Identification of PD-1 expression and immune infiltration-associated genes

DAVID (version 6.8) was used to investigate the involved Gene Ontology (GO)-biological processes and KEGG pathways of overlapping genes associated with PD-1 expression and immune infiltration.

### Prognostic model establishment and validation

Univariate and multivariable Cox and LASSO regression analyses were performed to select prognostic genes for modeling. GSE131769, GSE86166 (https://www.ncbi.nlm.nih.gov/geo/) and METABRIC (http://www.cbioportal.org) datasets were used to validate the prognostic model. The sample risk scores were calculated per the formula. Risk score = βgene1 × exprgene1 + βgene2 × exprgene2+ … + βgenen × exprgenen. Patients were assigned into two different risk groups based on the median risk score values. The overall survival, risk score distribution, and gene expression pattern within these two risk groups were analyzed using the survminer package (version 0.4.3) in R. The distributions of Riskscores of samples grouped by clinical and demographic factors, including age, race, tumor stage, pathologic T/N/M stage, and estrogen receptor (ER), her2 receptor, and progesterone receptor (PR) status were analyzed.

### Statistical analysis

*P* value or adjusted *P* value < 0.05 was considered as statistically significant in the whole study.

The detailed methods were presented in Methods supplement.

## Results

### PD-1 is prognosis-related in BC

**Fig. 1:**
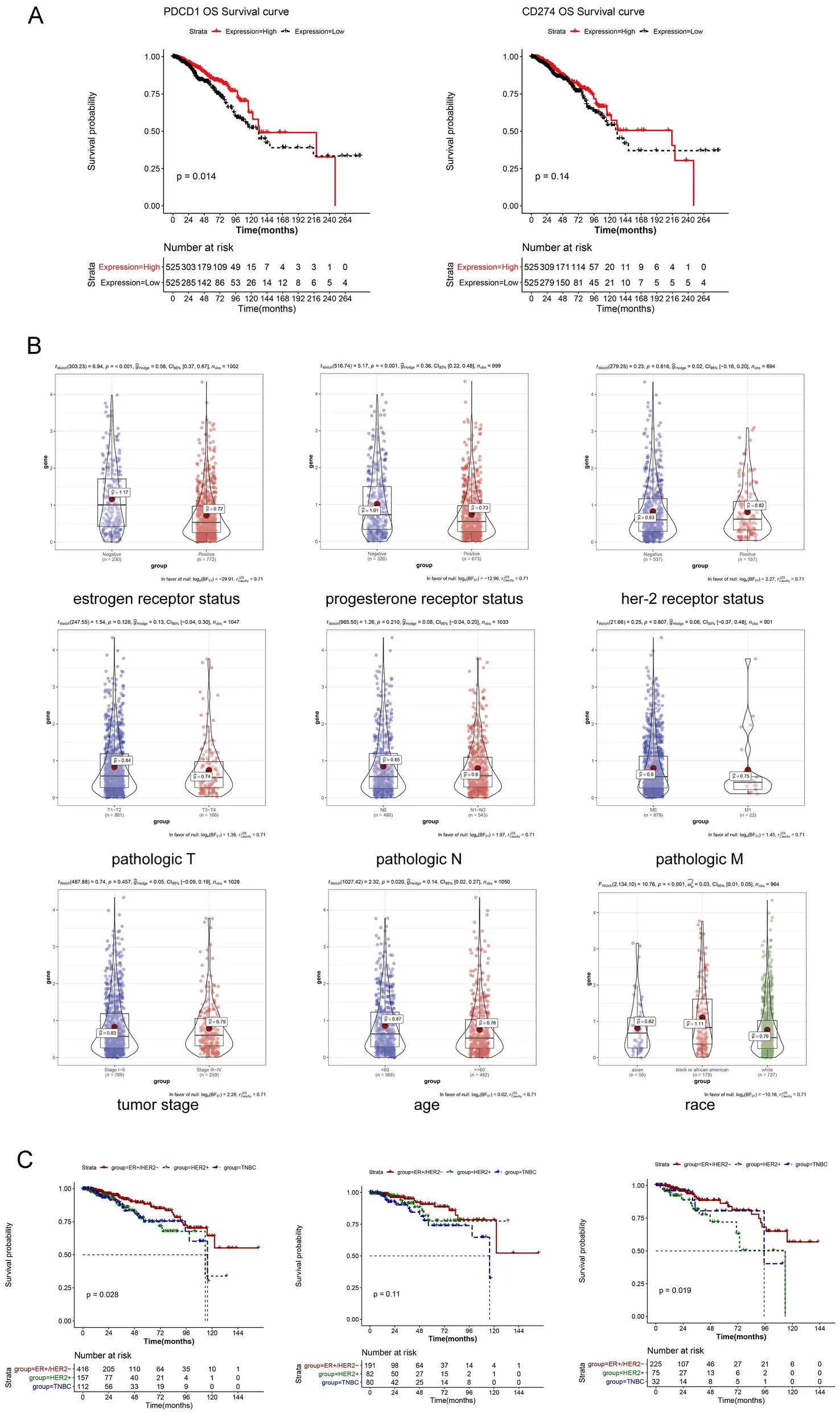
PD-1 expression association with survival and clinical factors. **A** The K–M survival curves show the prognostic value of PD-1 and PD-L1 expression for breast cancer patients. **B** The violin plots show the differences between PD-1 expression status and age, race, tumor stage, pathologic T/N/M, and estrogen receptor, her2 receptor, and progesterone receptor status. **C** The K–M survival curves show the prognostic value of PD-1 expression among three breast cancer subtypes

In TCGA dataset, the PD-1 and PD-L1 prognostic values were evaluated using the survival analysis. As shown in Fig. 1A, patients with high PD-1 tended to exhibit better survival rates than those with low PD-1 (*P* = 0.014), while patients with high PD-L1 tended to exhibit no significant difference in survival rates than those with low PD-L1 (*P* = 0.14).

### PD-1 expression with clinical factors

To investigate the associations between the PD-1 expression and clinical factors, the patients were divided into sub-groups based on different clinical factors, followed by the comparison of differences with the PD-1 expression. The results showed that the PD-1 expression exhibited no difference between the sub-groups divided by tumor stage, pathologic T/N/M, and her2 receptor status. PD-1 expression was associated with age, race, and PR and ER status (Fig. 1B). Patients aged < 60 years had high PD-1 expression in comparison with those aged ≥ 60 years (*P* = 0.020). Black or African American patients had high PD-1 expression in comparison with Asian patients (*P* < 0.001). ER-negative and PR-negative patients had higher PD-1 expression than those with ER-positive and PR-positive status (*P* < 0.01).

### Survival analysis of three BC subtypes

As shown in Fig. 1C, there was no significant difference in the survival of three BC subtypes in high PD-1 expression group (*P* = 0.11), while there was significant difference in the survival of three BC subtypes in low PD-1 expression (*P* = 0.019). And the prognosis of HER2+ subtype was poor. Among all samples, there were significant differences in survival among three BC subtypes (*P* = 0.028). TNBC showed the worst prognosis in high PD-1 expression group, while HER2+ showed poor prognosis in low PD-1 expression group. ER+ /HER2− type has a better prognosis in both high and low PD-1 expression groups.

### PD-1 expression-associated pathways

**Fig. 2:**
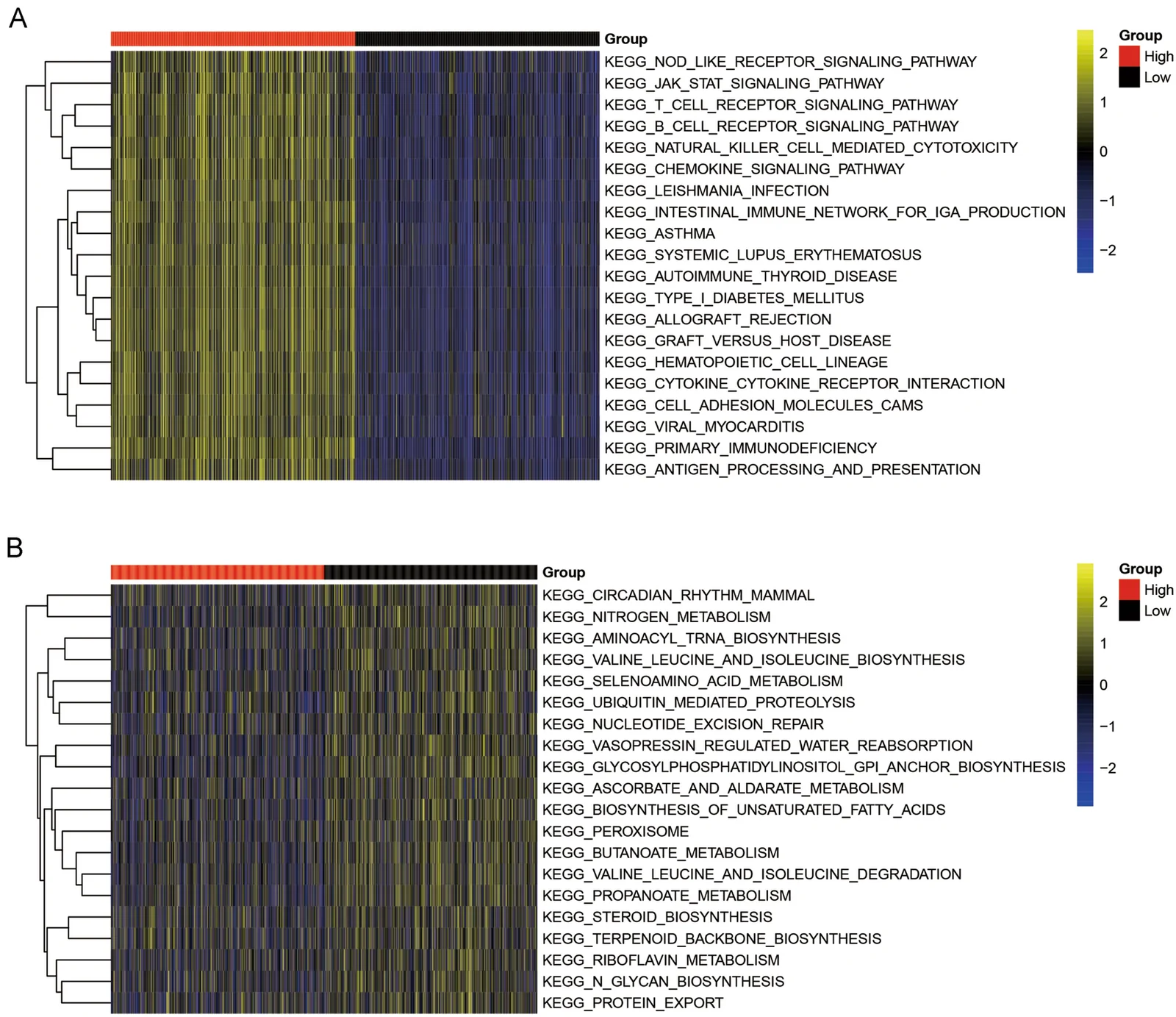
Results of gene set variation analysis. The heatmaps show the significantly enriched KEGG pathways in the (**A**) high- and (**B**) low-PD-1 expression groups

GSVA was used to investigate the differences in the KEGG pathways between the high- and low-PD-1 expression groups. We screened a total of 119 pathways showing significant differences. Figure 2 displays the top 20 pathways in these two expression groups. Multiple immune-associated pathways were enriched in the high PD-1 expression group, such as B cell and T cell receptor signaling pathways, natural killer cell-mediated cytotoxicity, or chemokine signaling pathway (Fig. 2A). Various biosynthesis- and metabolism-associated pathways were visibly enriched in the low-PD-1 expression group, including nitrogen, riboflavin, steroid, butanoate, ascorbate, and aldarate metabolism, as well as steroid, valine, leucine, and isoleucine biosynthesis (Fig. 2B).

### PD-1 expression-associated genes

**Fig. 3:**
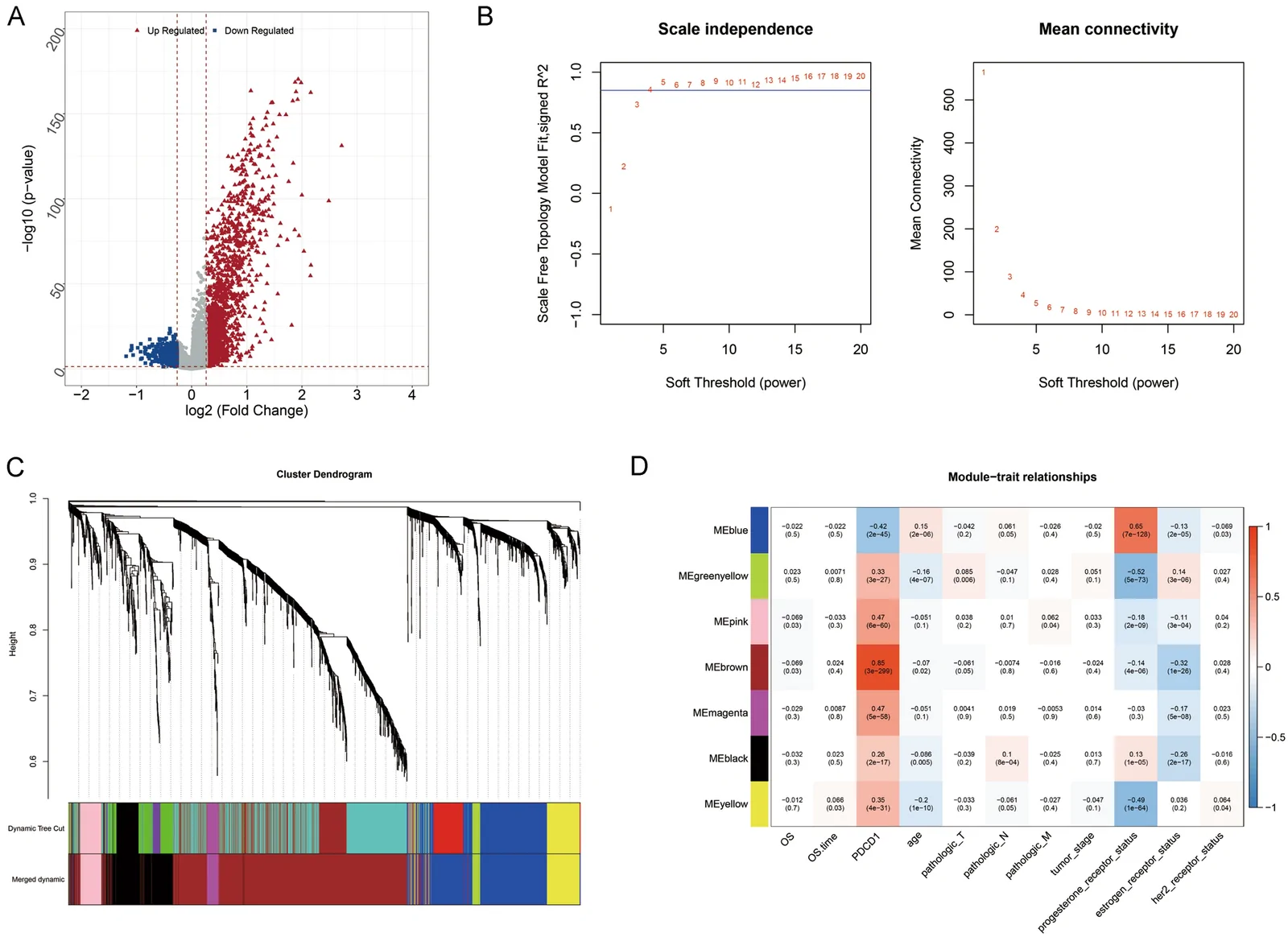
Identification of PD-1 expression-associated genes. **A** Volcano plot showing the differentially expressed genes between the high- vs. low-PD-1 expression groups. The red and blue dots represent the up- and down-regulated genes, respectively. **B** Calculation of soft-threshold (power) in weighted gene correlation network analysis (WGCNA). **C** Cluster dendrogram generated by hierarchical clustering based on gene dissimilarity measures. **D** Correlations analysis for gene modules and traits

A total of 2379 DEGs were screened between the high- and low-PD-1 expression groups, and we observed that the majority of the genes (*n* = 1861; 78.2% vs. *n* = 518; 21.8%) were up-regulated in the high PD-1 expression group (Fig. 3A, Table S1). These 2379 DEGs were analyzed by WGCNA. A soft-threshold power of four was selected to balance the relations between mean connectivity and scale independence (Fig. 3B). Based on clustering and dynamic pruning, genes with high correlations were aggregated into modules, resulting in a total of seven modules (Fig. 3C). Figure 3D shows the associations between the clinical factors and modules. The PD-1 expression showed strong positive and negative correlations with the brown and blue modules, respectively. These two modules were considered the key modules, further showing correlations with the PR and ER status. The brown and blue modules contained 1132 and 563 genes, respectively. These 1695 genes were considered PD-1 expression-associated genes.

### Identification of immune infiltration-associated genes

**Fig. 4:**
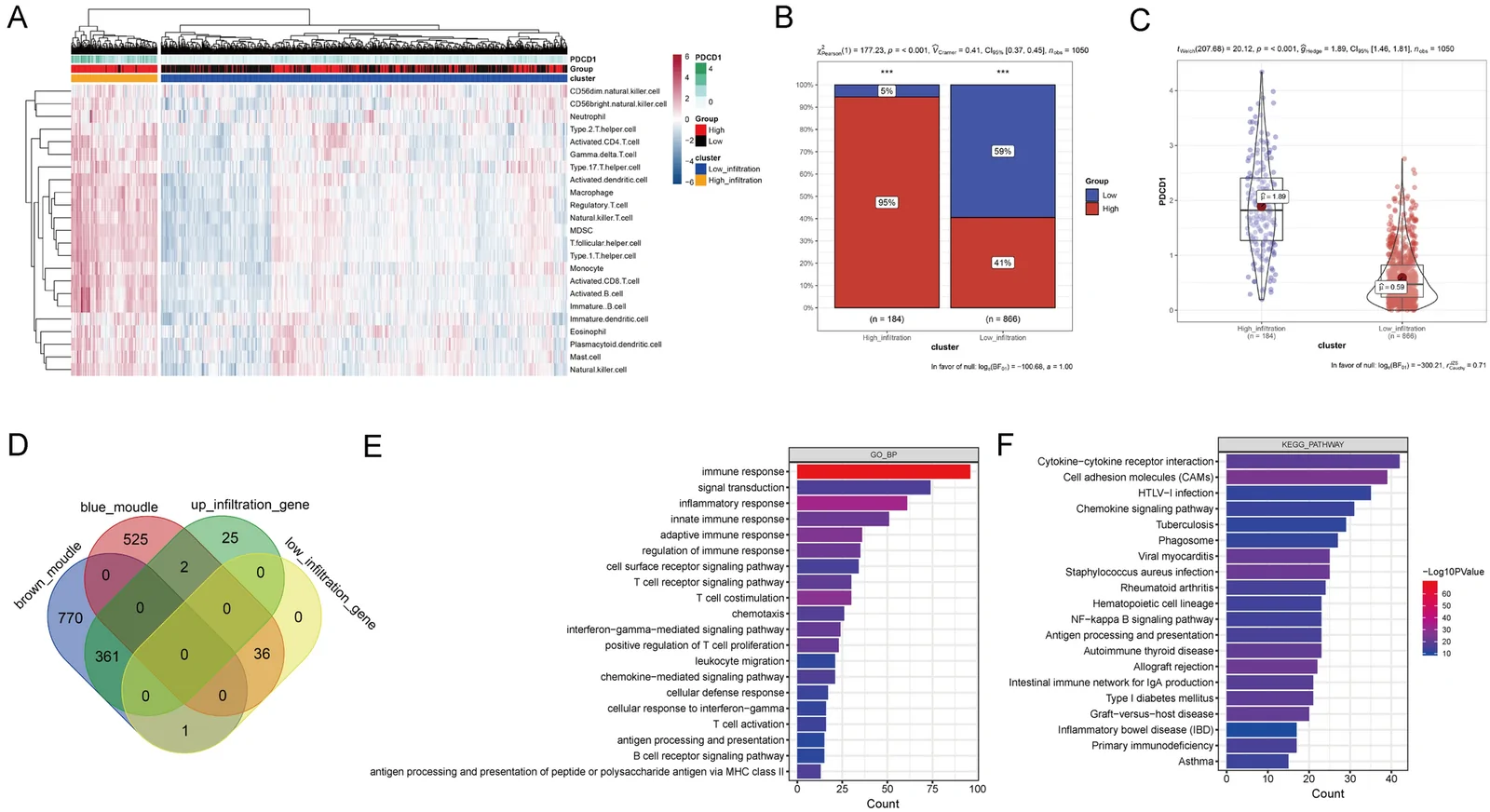
Immune infiltration- and PD-1 expression-associated genes. **A** Heatmap showing the enrichment score of 23 immune cell infiltration cases. **B** Histogram shows the proportion of patients with high- and low-PD-1 expression status in different infiltration groups. **C** Violin plots showing the PD-1 expression status differences between the high- and low-infiltration groups. **D** Venn plot showing the overlapping genes between the PD-1 expression-associated genes (genes in two WGCNA modules) and immune infiltration-associated genes (differentially expressed genes in the high- and low-infiltration groups). **E** Top 20 significantly enriched GO-biological processes and **F** KEGG pathways

The 23 immune cell infiltration abundance cases were evaluated using the enrichment score calculated by ssGSEA. Next, the samples were assigned into high- and low-infiltration groups (Fig. 4A). Samples with high PD-1 expression were mainly clustered in the high-infiltration group, and most samples with PD-1 low expression were clustered in the low-infiltration group. The high- and low-infiltration groups showed significantly different proportions of patients with high PD-1 expression (95 vs. 41%) and patients with low PD-1 expression (5 vs. 59%) (Fig. 4B). The patients in the high-infiltration group exhibited a significantly higher PD-1 expression level (Fig. 4C).

In addition, 429 DEGs were screened between the high- and low-immune infiltration groups (Table S2). These 429 genes were considered immune infiltration-associated genes. Most genes (*n* = 392, 91.4%) were up-regulated in the high-infiltration group.

### Immune infiltration- and PD-1 expression-associated genes

The PD-1 expression- (screened by WGCNA) and immune infiltration-associated genes were merged to identify genes associated with both immune infiltration and PD-1 expression. The Venn analysis resulted in 397 overlapping genes, comprising 361 up-regulated and 36 down-regulated genes (Fig. 4D, Table S3).

Next, we carried out a functional enrichment analysis for these overlapping genes. We found a significant enrichment of a total of 254 GO-biological processes and 45 KEGG pathways. Figure 4E–F shows the top 20 GO-biological processes and pathways, indicating that these genes were mainly implicated in immune-related biological processes, containing immune, inflammatory, innate immune, and adaptive immune responses, as well as T cell co-stimulation, proliferation, or activation. The top 20 pathways included cytokine–cytokine receptor interaction, chemokine signaling pathway, cell adhesion molecules, and antigen processing and presentation.

### Prognostic model

**Fig. 5:**
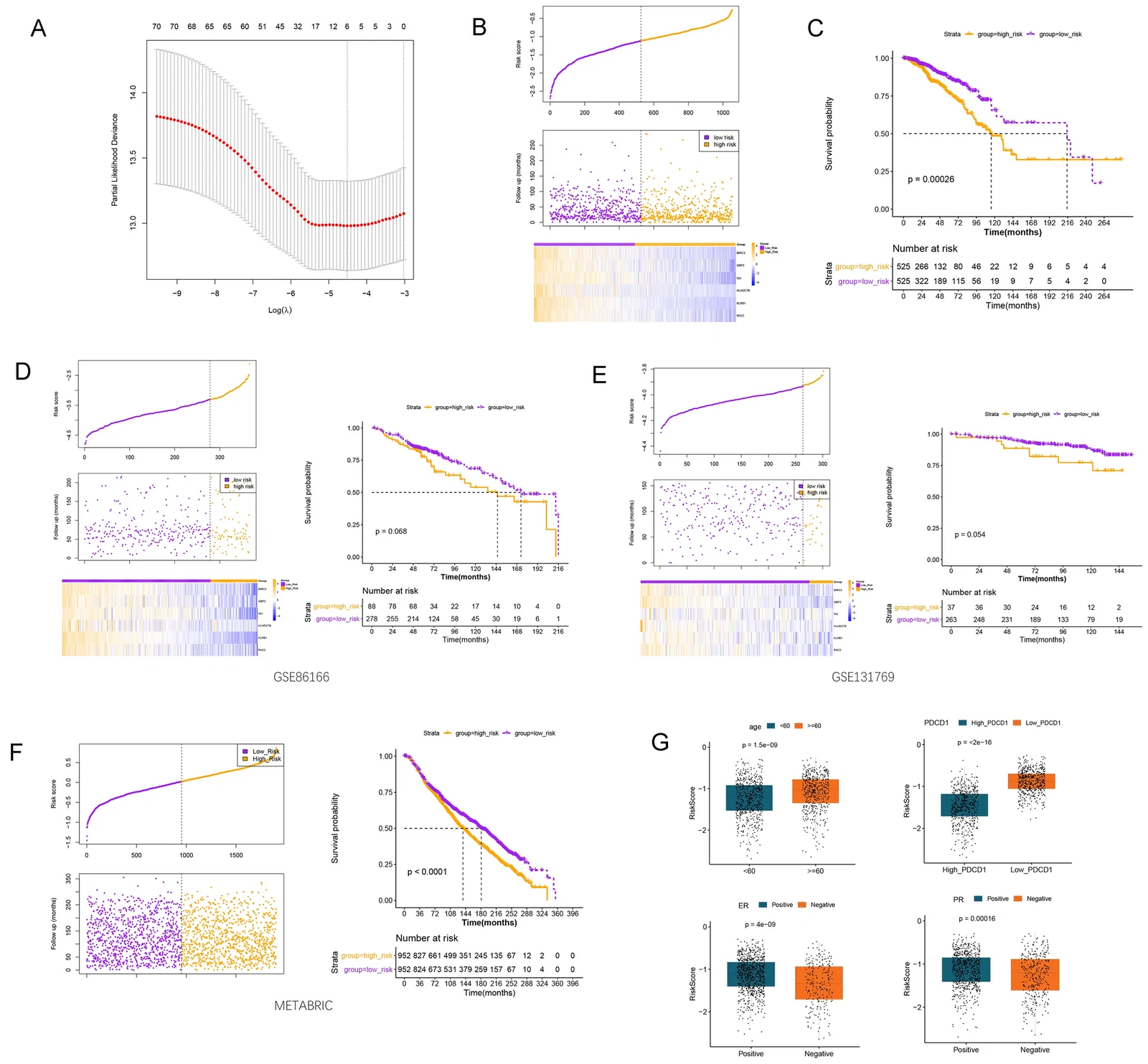
Prognostic model establishment and validation. **A** Selection of the optimal parameter lambda.min in the LASSO regression model. The dotted curve on the left and the right indicate lambda.min and lambda.1se, respectively. The model constructed based on lambda.1se is the simplest as a small number of genes were involved. The model constructed based on lambda.min is more accurate, involving a larger number of genes. For the horizontal axis, the upper numbers represent the numbers of variables corresponding to different *λ* values at bottom. The vertical axis represents partial likelihood deviance. **B** Risk score distribution between high- and low-risk groups. The risk score can assign patients into high- and low-risk groups (upper). The survival status of patients in the high- and low-risk groups. The vertical and horizontal axes represent the follow-up (months) and the risk score (middle), respectively. Heatmaps of gene expression patterns with risk scores from low to high. The vertical and horizontal axes represent the six genes and the risk score, respectively. The expression of these six genes gradually decreased with the risk score from low to high (bottom). **C** K–M curves showing the overall survival between the high- and low-risk groups. **D** Prognostic model validation in the GSE86166 dataset. **E** Prognostic model validation in the GSE131769 dataset. **F** Prognostic model validation in the METABRIC dataset. **G** The association between clinical parameters (age, PD1 expression, ER and PR status) and risk scores were analyzed

Among the 397 immune infiltration- and PD-1 expression-associated genes, our univariate Cox regression analysis identified 70 genes correlating with prognosis (*P* < 0.01). Based on the optimal parameter lambda.min, the LASSO regression model was used to select the most valuable prognostic genes from these 70 genes. As a result, six genes were selected, namely IRC3, GBP2, IGJ, KLHDC7B, KLRB1, and RAC2 (Fig. 5A). Next, we performed multivariable Cox regression analysis for the calculation of the prognostic correlation coefficient for these six genes (Table S3). Based on the prognostic correlation coefficient and the expression value, we established a six-gene prognostic model. After calculating the risk score for each sample, we assigned them all into two different risk groups according to the median risk score. The expression of these six genes gradually decreased with a risk score from low to high (Fig. 5B). Patients in the high-risk group were associated with adverse survival compared to those in the low-risk group (Fig. 5C).

### External validation of the prognostic model

The GSE131769, GSE86166 and METABRIC datasets for the external validation of the prognostic model. Similarly, the risk score calculated using the six-gene prognostic model could classify the patients into two risk groups. The expression of these six genes gradually decreased with risk scores from low to high. Moreover, the patients in the high-risk group tended to exhibit worse survival compared to those in the low-risk group. The survival differences showed no statistical significance, with *P* = 0.054, *P* = 0.068 in the GSE131769 and GSE86166 (Fig. 5D–E), respectively. However, the survival differences showed statistical significance, with *P* < 0.0001 in METABRIC datasets (Fig. 5F). There were significant differences in RiskScore between under- and over-60-year groups (*P* < 0.01), PDCD1 high- and low-expression groups (*P* < 0.01), ER-positive and -negative groups (*P* < 0.01), and PR-positive and -negative groups (*P* = 0.00016) (Fig. 5G). There were no significant differences in different race, tumor stage, pathologic T/N/M stage, and her2 receptor groups (*P* > 0.05).

## Discussion

PD-1/PD-L1 are important co-stimulatory molecules involved in the regulation of immune responses. When PD-L1 and PD-1 interact, the immune response of tumor T lymphocytes is suppressed, so that tumor cells can escape the immune response [10]. The anti-PD-1/PD-L1 antibodies have been approved by the FDA in an unprecedentedly fast manner to treat various cancers. Recently, the evaluation of the PD-1/PD-L1 blockade in BC is ongoing, especially in TNBC, showing promising results [17]. Therefore, we investigated the potential underlying PD-1/PD-L1 regulatory mechanisms and their prognostic value in BC.

Using the data in TCGA, the association between PD-1/PD-L1 expression status, patient survival, and PD-1/PD-L1 expression status with clinical factors was investigated. Patients with high PD-1 expression demonstrated a favorable prognosis compared to those with low PD-1 expression (*P* = 0.014), while patients with high and low PD-L1 expression showed no significant difference in survival time. Moreover, Matikas et al. [24], Jiang et al. [25] suggested that the expression of PD-1 protein correlated with improved overall survival for BC patients. These results suggested that high PD-1 expression might be a favorable prognostic marker in BC. However, we found that ER- and PR-negative patients had high PD-1 expression compared with patients displaying ER- and PR-positive status (*P* < 0.01). Similar results were also found in a previous study [25], which indicated that ER-negative (4.35 vs. 1.82, *P* < 0.001) and PR-negative (3.47 vs. 1.86, *P* < 0.001) BC patients had higher PD-1 expression than that in ER-positive and PR-positive patients. This result suggested that PD-1 expression varies among the different BC subtypes and might explain why PD-1/PD-L1 blockade showed promising results, especially in TNBC. Meanwhile, patients who aged < 60 years had high PD-1 expression in comparison with those aged ≥ 60 years (*P* = 0.020). Black or African American patients had high PD-1 expression in comparison with Asian patients (*P* < 0.001). It indicated that age and race should be considered when using PD1 therapy.

To investigate the potential underlying mechanism of survival difference between high and low PD-1 expression, GSVA and immune infiltration analysis were performed. GSVA revealed that various immune-associated pathways were enriched in the high PD-1 expression group, such as B cell and T cell receptor signaling pathways, natural killer cell-mediated cytotoxicity, or chemokine signaling pathway. Moreover, the immune infiltration analysis revealed that patients in the high-infiltration group showed a significantly elevated PD-1 expression level. These results suggested that the improved survival of patients with higher PD-1 expression levels might be mainly attributed to increased immune infiltration and the activation of immune response-associated genes.

There were 1861 up-regulated DEGs and 518 down-regulated DEGs between high and low PD1 expression groups. WGCNA analysis of these DEGs showed that positive and negative PD1 expression were significantly correlated with age, PR and ER status. It indicated that age and BC subtypes should be taken into full consideration when using PD1 therapy again. Later, 429 DEGs were found between the high- and low- immune infiltration groups. Then, 397 genes associated with both immune infiltration and PD-1 expression were screened and 70 genes of among them correlated with the prognosis. These genes were mainly implicated in immune-related biological processes, suggesting that prognosis correlated with immune response. LASSO regression model was used to select the most valuable prognostic genes, identifying six genes, namely IRC3, GBP2, IGJ, KLHDC7B, KLRB1, and RAC2. IRC3 is involved in mitochondrial genome stability maintenance [26]. Genomic instability is a hallmark of tumorigenesis, mainly associated with the accumulation of DNA damage [27]. The dysregulation of IRC3 expression might affect DNA replication and repair in BC. GBP2 encodes a guanylate-binding protein induced by interferon-γ [28]. Increased GBP2 expression in BC showed correlations with improved survival and tumor-infiltrating T cell [29]. GBP2 could suppress cell metastasis and mitochondrial fission in BC cells both in vitro and in vivo [30]. High IGJ expression showed correlations with improved overall early BC patient survival [31]. Tumors with high proportion of Ki67-positive cells and ductal tumors exhibited the highest KLHDC7B expression, suggesting that KLHDC7B could be a biomarker for poorly differentiated BC [32]. KLHDC7B may inhibit BC tumorigenesis by regulating interferon signals [33]. KLRB1 is expressed by NK cells and encodes the NK cell CD161 receptor, involving regulation of NK cell function [34]. NK cells are lymphocytes that modulate cytotoxicity and secrete cytokines following immune stimulation. KLRB1 expression was inhibited in tumor tissues, and it potentiated be a prognostic biomarker in carcinoma. RAC2 is expressed solely in hematopoietic cells that play an important role in neutrophils and lymphocytes [35]. High RAC2 expression was associated with adverse survival, suggesting its potential as a promising prognostic marker [36].

Based on the 6 genes, Riskscore model was constructed. External datasets, including GSE131769, GSE86166 and METABRIC were used for detection the Riskscore. There was no significant difference in survival between the high- and low-risk groups in GSE131769 and GSE86166 datasets. However, there was significant difference in survival between the high- and low-risk groups in METABRIC, and the high-risk group had worse prognosis. The difference in verification results between GSE131769, GSE86166 databases and METABRIC database is probably due to the insufficient sample size in GSE131769 and GSE86166 databases. The validation result of METABRIC database that owns large sample size is more accurate and convincing. Immune response played important role in the anti-tumor process. These six immune response-associated genes correlated with T cells, NK cells, interferon, etc. Up-regulated or down-regulated expressions of these six genes might affect the function of immune cells and secretion of immune-related molecules. So the prognostic model established based on these six immune response-associated genes could predict overall BC patient survival, suggesting their prognostic value in BC.

In conclusion, we investigated PD-1 expression status with patient survival and clinical factors in BC. High PD-1 expression could predict a favorable BC prognosis. PD-1 expression levels varied among different subtypes, with high PD-1 expression in ER- and PR-negative patients. The favorable patient survival with high PD-1 expression might be mainly attributed to the increased immune infiltration and activation of immune response-associated genes. We identified six genes, namely IRC3, GBP2, IGJ, KLHDC7B, KLRB1, and RAC2, associated with both immune infiltration and PD-1 expression as key prognostic genes in BC. The prognostic model based on these six genes could predict overall BC patient survival. These genes might be potential prognostic biomarkers to predict the response to anti-PD-1 therapy.

## Supplementary Information

Below is the link to the electronic supplementary material.

Supplementary file1 Study workflow (TIF 2737 KB)

Supplementary file2 List of differentially expressed genes between the high- and low-PD-1 expression groups (XLS 376 KB)

Supplementary file3 List of differentially expressed genes between the high- and low-immune infiltration groups (XLSX 50 KB)

Supplementary file4 Venn analysis of screening for genes associated with both immune infiltration and PD-1 expression (XLS 41 KB)

Supplementary file5 Prognostic correlation coefficient calculated using the multivariable Cox regression analysis (DOC 29 KB)

## Data Availability

The datasets generated during the current study are not publicly available but obtained from corresponding authors on reasonable request.

## Abbreviations

PD-1: Programmed cell death-1
BC: Breast cancer
GSVA: Gene set variation analysis
TNBC: Triple-negative BC
WGCNA: Weighted gene correlation network analysis
K–M: Kaplan–Meier
ER: Estrogen receptor
PR: Progesterone receptor
DEGs: Differentially expressed genes
ssGSEA: Single sample GSEA
