# Supplementary figures and images for: Prognostic model based on six PD-1 expression and immune infiltration-associated genes predicts survival in breast cancer
Source: Breast Cancer. 2022 Mar 1;29(4):666–76. doi: 10.1007/s12282-022-01344-2 (PMC9226094; doi:10.1007/s12282-022-01344-2)

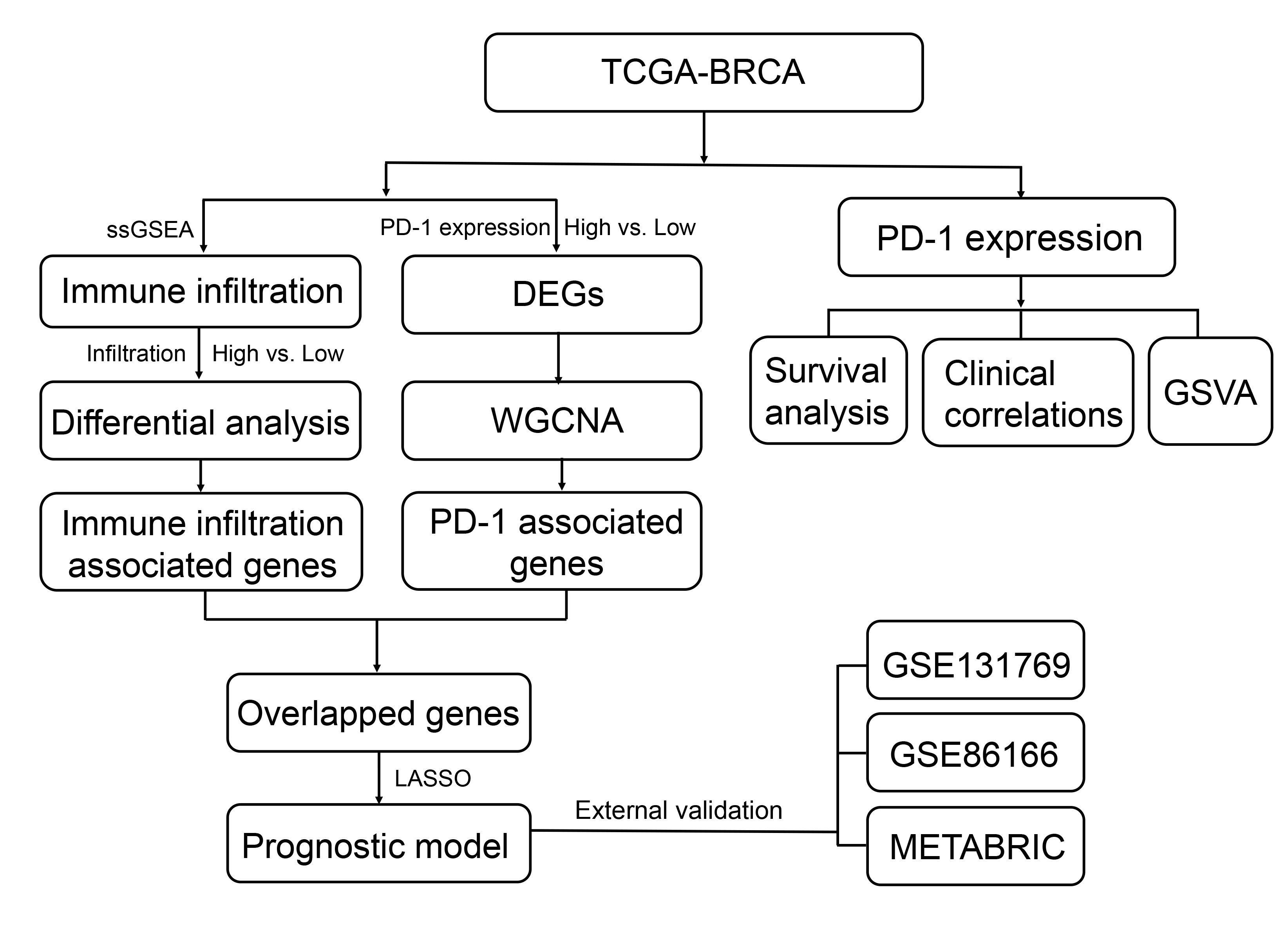

Supplement: Supplementary file 1 — Supplementary file1 Study workflow (TIF 2737 KB) [file 12282_2022_1344_MOESM1_ESM.tif]
