# Supplementary material for: Prognostic model based on six PD-1 expression and immune infiltration-associated genes predicts survival in breast cancer
Source: Breast Cancer. 2022 Mar 1;29(4):666–76. doi: 10.1007/s12282-022-01344-2 (PMC9226094; doi:10.1007/s12282-022-01344-2)
Supplement: Supplementary file 5 — Supplementary file5 Prognostic correlation coefficient calculated using the multivariable Cox regression analysis (DOC 29 KB) [file 12282_2022_1344_MOESM5_ESM.doc]

Table S1. prognostic correlation coefficient calculated by multivariable Cox regression analysis

| gene | coef |
| --- | --- |
| BIRC3 | -0.033217 |
| GBP2 | -0.053711 |
| IGJ | -0.057196 |
| KLHDC7B | -0.109943 |
| KLRB1 | -0.22394 |
| RAC2 | -0.035129 |
